# Supplementary material for: COVID-19: The Disease, the Immunological Challenges, the Treatment with Pharmaceuticals and Low-Dose Ionizing Radiation
Source: Cells. 2021 Aug 27;10(9):2212. doi: 10.3390/cells10092212 (PMC8470324; doi:10.3390/cells10092212)
Supplement: Supplementary file 1 [file cells-10-02212-s001.zip › Table S1_vaccine landscape.pdf]

Table S1. The development of COVID-19 vaccines in clinical trials (adjusted from WHO document) [1].

ID	Vaccine platform description	Type of candidate vaccine	Dose number	Schedule	Route of administration	Developers	Phase
1	Inactivated virus	CoronaVac; SARS-CoV-2 vaccine (inactivated)	2	Day 0 + 14	IM	Sinovac Research and Development Co., Ltd	4
2	Inactivated virus	Inactivated SARS-CoV-2 vaccine (Vero cell)	2	Day 0 + 21	IM	Sinopharm + China National Biotec Group Co + Wuhan Institute of Biological Products	3
3	Inactivated virus	Inactivated SARS-CoV-2 vaccine (Vero cell), vaccine name BBIBP-CorV	2	Day 0 + 21	IM	Sinopharm + China National Biotec Group Co + Beijing Institute of Biological Products	3
4	Viral vector (Non-replicating)	ChAdOx1-S - (AZD1222) (Covishield)	1-2	Day 0 + 28	IM	AstraZeneca + University of Oxford	4
			1-2	Day 0 + 28	IN	University of Oxford	1
5	Viral vector (Non-replicating)	Recombinant novel coronavirus vaccine (Adenovirus type 5 vector)	1	Day 0	IM	CanSino Biological Inc./Beijing Institute of Biotechnology	3
6	Viral vector (Non-replicating)	Gam-COVID-Vac Adeno-based (rAd26-S+rAd5-S)	2	Day 0 + 21	IM	Gamaleya Research Institute ; Health Ministry of the Russian Federation	3
7	Viral vector (Non-replicating)	Ad26.COV2.S	1-2	Day 0 or Day 0 +56	IM	Janssen Pharmaceutical	3
8	Protein subunit	SARS-CoV-2 rS/Matrix M1-Adjuvant (Full length recombinant SARS CoV-2 glycoprotein nanoparticle vaccine adjuvanted with Matrix M)	2	Day 0 + 21	IM	Novavax	3
9	RNA based vaccine	mRNA -1273 mRNA-1283	2	Day 0 + 28	IM	Moderna + National Institute of Allergy and Infectious Diseases (NIAID)	4
10	RNA based vaccine	BNT162b2 (3 LNP-mRNAs), also known as "Comirnaty"	2	Day 0 + 21	IM	Pfizer/BioNTech + Fosun Pharma	4
11	Protein subunit	Recombinant SARS-CoV-2 vaccine (CHO Cell)	2-3	Day 0 + 28 or Day 0 + 28 + 56	IM	Anhui Zhifei Longcom Biopharmaceutical + Institute of Microbiology, Chinese Academy of Sciences	3
12	RNA based vaccine	CVnCoV Vaccine	2	Day 0 + 28	IM	CureVac AG	3

13	Inactivated virus	SARS-CoV-2 vaccine (vero cells)	2	Day 0 + 28	IM	Institute of Medical Biology + Chinese Academy of Medical Sciences	3
14	Inactivated virus	QazCovid-in® - COVID-19 inactivated vaccine	2	Day 0 + 21	IM	Research Institute for Biological Safety Problems, Rep of Kazakhstan	3
15	DNA based vaccine	INO-4800+electroporation	2	Day 0 + 28	ID	Inovio Pharmaceuticals + International Vaccine Institute + Advaccine (Suzhou) Biopharmaceutical Co., Ltd	2/3
16	DNA based vaccine	AG0301-COVID19	2	Day 0 + 14	IM	AnGes + Takara Bio + Osaka University	2/3
17	DNA based vaccine	nCov vaccine	3	Day 0 + 28 + 56	ID	Zydus Cadila	3
18	DNA based vaccine	GX-19N	2	Day 0 + 28	IM	Genexine Consortium	1/2
19	Inactivated virus	Whole-Virion Inactivated SARS-CoV-2 Vaccine (BBV152)	2	Day 0 + 14	IM	Bharat Biotech International Limited	3
20	Protein subunit	KBP-COVID-19 (RBD-based)	2	Day 0 + 21	IM	Kentucky Bioprocessing Inc.	1/2
21	Protein subunit	VAT00002: SARS-CoV-2 S protein with adjuvant	2	Day 0 + 21	IM	Sanofi Pasteur + GSK	3
22	RNA based vaccine	ARCT-021	ND	ND	IM	Arcturus Therapeutics	2
23	Virus like particle	RBD SARS-CoV-2 HBsAg VLP vaccine	2	Day 0 + 28	IM	Serum Institute of India + Accelagen Pty + SpyBiotech	1/2
24	Inactivated virus	Inactivated SARS-CoV-2 vaccine (Vero cell)	1,2 or 3	ND	IM	Beijing Minhai Biotechnology Co	3
25	Viral vector (Non-replicating)	GRAd-COV2 (Replication defective Simian Adenovirus (GRAd) encoding S)	1	Day 0	IM	ReiThera + Leukocare + Univercells	2/3
26	Viral vector (Non-replicating)	VXA-CoV2-1 Ad5 adjuvanted Oral Vaccine platform	2	Day 0 + 28	Oral	Vaxart	1
27	Viral vector (Non-replicating)	MVA-SARS-2-S	2	Day 0 + 28	IM	University of Munich (Ludwig-Maximilians)	1
28	Protein subunit	SCB-2019 + AS03 or CpG 1018 adjuvant plus Alum adjuvant (Native like Trimeric subunit Spike Protein vaccine)	2	Day 0 + 21	IM	Clover Biopharmaceuticals Inc./GSK/Dynavax	2/3
29	Protein subunit	COVAX-19® Recombinant spike protein + adjuvant	1	Day 0 + 21	IM	Vaxine Pty Ltd.	1
30	Protein subunit	MVC-COV1901 (Spike-2P protein + adjuvant CpG 1018)	2	Day 0 + 28	IM	Medigen Vaccine Biologics + Dynavax + National Institute of Allergy and	2

						Infectious Diseases (NIAID)	
31	Protein subunit	FINLAY-FR1 anti-SARS-CoV-2 Vaccine (RBD + adjuvant)	2	Day 0 + 28	IM	Instituto Finlay de Vacunas	1/2
32	Protein subunit	FINLAY-FR-2 anti-SARS-CoV-2 Vaccine (RBD chemically conjugated to tetanus toxoid plus adjuvant)	2	Day 0 + 28	IM	Instituto Finlay de Vacunas	3
33	Protein subunit	EpiVacCorona (EpiVacCorona vaccine based on peptide antigens for the prevention of COVID-19)	2	Day 0 + 21	IM	Federal Budgetary Research Institution State Research Center of Virology and Biotechnology "Vector"	3
34	Protein subunit	RBD (baculovirus production expressed in Sf9 cells) Recombinant SARS-CoV-2 vaccine (Sf9 Cell)	2	Day 0 + 28	IM	West China Hospital + Sichuan University	2
35	Protein subunit	IMP CoVac-1 (SARS-CoV-2 HLA-DR peptides)	1	Day 0	SC	University Hospital Tuebingen	1
36	Protein subunit	UB-612 (Multitope peptide based S1-RBD-protein based vaccine)	2	Day 0 + 28	IM	Vaxxinity	3
37	Viral vector (Replicating)	DelNS1-2019-nCoV-RBD-OPT1 (Intranasal flu-based-RBD)	2	Day 0 + 28	IN	University of Hong Kong, Xiamen University and Beijing Wantai Biological Pharmacy	2
38	RNA based vaccine	LNP-nCoVsaRNA	2	ND	IM	Imperial College London	1
39	RNA based vaccine	SARS-CoV-2 mRNA vaccine (ARCoV)	2	Day 0 + 14 or Day 0 + 28	IM	Academy of Military Science (AMS), Walvax Biotechnology and Suzhou Abogen Biosciences	3
40	Virus like particle	Coronavirus-Like Particle COVID-19 (CoVLP)	2	Day 0 + 21	IM	Medicago Inc.	2/3
41	Viral vector (Replicating) + APC	Covid-19/aAPC vaccine. The Covid-19/aAPC vaccine is prepared by applying lentivirus modification with immune modulatory genes and the viral minigenes to the artificial antigen presenting cells (aAPCs).	3	Day 0 + 14 + 28	SC	Shenzhen Geno-Immune Medical Institute	1
42	Viral vector (Non-replicating) + APC	LV-SMENP-DC vaccine. Dendritic cells are modified with lentivirus vectors	1	Day 0	SC & IV	Shenzhen Geno-Immune Medical Institute	1/2

		expressing Covid-19 minigene SMENP and immune modulatory genes. CTLs are activated by LV-DC presenting Covid-19 specific antigens.					
43	Protein subunit	AdimrSC-2f (recombinant RBD +/- Aluminium)	ND	ND	ND	Adimmune Corporation	1
44	DNA based vaccine	Covigenix VAX-001 - DNA vaccines + proteo-lipid vehicle (PLV) formulation	2	Day 0 + 14	IM	Entos Pharmaceuticals Inc.	1
45	DNA based vaccine	CORVax - Spike (S) Protein Plasmid DNA Vaccine	2	Day 0 + 14	ID	Providence Health & Services	1
46	RNA based vaccine	ChulaCov19 mRNA vaccine	2	Day 0 + 21	IM	Chulalongkorn University	1
47	DNA based vaccine	bacTRL-Spike oral DNA vaccine	1	Day 0	Oral	Symvivo Corporation	1
48	Viral vector (Non-replicating)	Human Adenovirus Type 5: hAd5 S+N vaccine (S-Fusion + N-ETSD). E2b- Deleted Adeno.	1-2	Day 0 + 21	SC or Oral	ImmunityBio, Inc	1/2
49	Viral vector (Non-replicating)	COH04S1 (MVA-SARS-2-S) - Modified vaccinia ankara (sMVA) platform + synthetic SARS-CoV-2	1-2	Day 0 + 28	IM	City of Hope Medical Center + National Cancer Institute	1
50	Viral vector (Replicating)	rVSV-SARS-CoV-2-S Vaccine	1	Day 0	IM	Israel Institute for Biological Research	1/2
51	Viral vector (Replicating) + APC	Dendritic cell vaccine AV-COVID-19. A vaccine consisting of autologous dendritic cells loaded with antigens from SARS-CoV-2, with or without GM-CSF	1	Day 0	IM	Aivita Biomedical, Inc. National Institute of Health Research and Development, Ministry of Health Republic of Indonesia	1/2
52	Live attenuated virus	COVI-VAC	1-2	Day 0 or Day 0 + 28	IN	Codagenix/Serum Institute of India	1
53	Protein subunit	CIGB-669 (RBD+AgnHB)	3	Day 0 + 14 + 28 or Day 0 +28 + 56	IN	Center for Genetic Engineering and Biotechnology (CIGB)	1/2
54	Protein subunit	CIGB-66 (RBD+aluminium hydroxide)	3	Day 0 + 14 + 28 or Day 0 +28 + 56	IM	Center for Genetic Engineering and Biotechnology (CIGB)	3
55	Inactivated Virus	VLA2001	2	Day 0 + 21	IM	Valneva, National Institute for Health Research, United Kingdom	1/2
56	Protein subunit	BECOV2	2	Day 0 + 28	IM	Biological E. Limited	1/2

57	Viral vector (Replicating)	AdCLD-CoV19 (adenovirus vector)	1	Day 0	IM	Cellid Co., Ltd.	1/2
58	DNA based vaccine	GLS-5310	2	Day 0 + 56 or Day 0 + 84	ID	GeneOne Life Science, Inc.	1/2
59	Protein subunit	Recombinant Sars-CoV-2 Spike protein, Aluminum adjuvanted	2	Day 0 + 21	IM	Nanogen Pharmaceutical Biotechnology	1/2
60	Protein subunit	Recombinant protein vaccine S-268019 (using Baculovirus expression vector system)	2	Day 0 + 21	IM	Shionogi	1/2
61	Viral vector (Non-replicating)	AdCOVID, Adenovirus-based platform expresses the receptor-binding domain (RBD) of the Sars-Cov-2 spike protein	1-2	Day 0	IN	Altimmune, Inc.	1
62	Protein subunit	SARS-CoV-2-RBD-Fc fusion protein			SC or IM	University Medical Center Groningen + Akston Biosciences Inc.	1/2
63	Inactivated Virus	ERUCOV-VAC, inactivated virus	2	Day 0 + 21	IM	Erciyes University	2
64	Protein subunit	COVAC-1 and COVAC-2 sub-unit vaccine (spike protein) + SWE adjuvant	2	Day 0 + 28	IM	University of Saskatchewan	1/2
65	Protein subunit	GBP510, a recombinant surface protein vaccine with adjuvant AS03 (aluminium hydroxide)	2	Day 0 + 28	IM	SK Bioscience Co., Ltd. and CEPI	1/2
66	Protein subunit	Razi Cov Pars, recombinant spike protein	3	Day 0 + 21 +51	IM and IN	Razi Vaccine and Serum Research Institute	2
67	Inactivated Virus	COVID-19 inactivated vaccine	2	Day 0 + 14	IM	Shifa Pharmed Industrial Co	2/3
68	Protein subunit	MF59 adjuvanted SARS-CoV-2 Sclamp vaccine	2	Day 0 + 28	IM	The University of Queensland	1
69	DNA based vaccine	COVIGEN	2	Day 0 + 28	ID or IM	University of Sydney, Bionet Co., Ltd Technovalia	1
70	DNA based vaccine	COVID-eVax, a candidate plasmid DNA vaccine of the Spike protein			IM or<br>IM + electroporation	Takis + Rottapharm Biotech	1/2
71	Viral vector (Non-replicating)	BBV154, Adenoviral vector COVID-19 vaccine	1	Day 0	IN	Bharat Biotech International Limited	1
72	RNA based vaccine	PTX-COVID19-B, mRNA vaccine	2	Day 0 + 28	IM	Providence Therapeutics	1
73	Viral vector (Replicating)	NDV-HXP-S, Newcastle disease virus (NDV) vector	2	Day 0 + 28	IM	Mahidol University; The Government Pharmaceutical	1/2

		expressing the spike protein of SARS-CoV-2, with or without the adjuvant CpG 1018				Organization (GPO); Icahn School of Medicine at Mount Sinai	
74	RNA based vaccine	CoV2 SAM (LNP) vaccine. A self-amplifying mRNA (SAM) lipid nanoparticle (LNP) platform + Spike antigen		Day 0 + 28	IM	GlaxoSmithKline	1
75	Virus like particle	VBI-2902a. An enveloped virus-like particle (eVLP) of SARS-CoV-2 spike (S) glycoprotein and aluminum phosphate adjuvant.	2	Day 0 + 28	IM	VBI Vaccines Inc.	1/2
76	Protein subunit	SK SARS-CoV-2 recombinant surface antigen protein subunit (NBP2001) + adjuvanted with alum.	2	Day 0 + 28	IM	SK Bioscience Co., Ltd.	1
77	Viral vector (Non-replicating)	Chimpanzee Adenovirus serotype 68 (ChAd) and self-amplifying mRNA (SAM) vectors expressing spike alone, or spike plus additional SARS-CoV-2 T cell epitopes.	2-3	Day 0 + 14 + 28 or Day 0 +28 + 56 or Day 0 + 112	IM	Gritstone Oncology	1
78	RNA based vaccine	mRNA-1273.351. A lipid nanoparticle (LNP)-encapsulated mRNA-based vaccine that encodes for a full-length, prefusion stabilized S protein of the SARS-CoV-2 B.1.351 variant.	3	Day 0 or Day 0 + 28 or Day 56	IM	Moderna + National Institute of Allergy and Infectious Diseases (NIAID)	2
79	Protein subunit	SpFN (spike ferritin nanoparticle) uses spike proteins with a liposomal formulation QS21 (ALFQ) adjuvant.	2-3	Day 0 + 28 + 180	IM	Walter Reed Army Institute of Research (WRAIR)	1
80	Protein subunit	EuCorVac-19; A spike protein using the recombinant protein technology and with an adjuvant.	2	Day 0 + 21	IM	POP Biotechnologies and EuBiologics Co.,Ltd	1/2
81	Inactivated virus	Inactivated SARS-CoV-2 vaccine FAKHRAVAC (MIVAC)	2	Day 0 + 14 +/- 21	IM	Organization of Defensive Innovation and Research	1
82	Live attenuated virus	MV-014-212, a live attenuated vaccine that	3	Day 0 +/- 35	IN	Meissa Vaccines, Inc.	1

		expresses the spike (S) protein of SARS-CoV-2					
83	RNA based vaccine	MRT5500, an mRNA vaccine candidate	2	Day 0 + 21	IM	Sanofi Pasteur and Translate Bio	1/2
84	Virus like particle	SARS-CoV-2 VLP Vaccine	2	Day 0	SC	The Scientific and Technological Research Council of Turkey	1
85	Protein subunit	ReCOV: Recombinant two-component spike and RBD protein COVID-19 vaccine (CHO cell).	2	Day 0 + 21	IM	Jiangsu Rec-Biotechnology	1
86	RNA based vaccine	DS-5670a, mRNA vaccine	2		IM	Daiichi Sankyo Co., Ltd.	1/2
87	Inactivated Virus	Inactivated COVID-19 vaccine	2	Day 0 + 21	IM	Kocak Farma	1
88	Viral vector (Non-replicating)	COVIVAC. Newcastle Disease Virus (NDV) expressing membrane-anchored pre-fusion-stabilized trimeric SARS-CoV-2 S protein +/- adjuvant CpG 1018	2	Day 0 + 28	IM	Institute of Vaccines and Medical Biologicals, Vietnam	1/2
89	Viral vector (Non-replicating)	SC-Ad6-1, Adneviral vector vaccine	1-2	Day 0 +/- 21	IM	Tetherex Pharmaceuticals Corporation	1
90	Virus like particle	ABNCoV2 capsid virus-like particle (cVLP) +/- adjuvant MF59	2	Day 0 + 28	IM	Radboud University	1
91	Protein subunit	Recombinant SARS-CoV-2 Fusion Protein Vaccine (V-01)	2	Day 0 + 21	IM	Guangdong Provincial Center for Disease Control and Prevention/Gaozhou Center for Disease Control and Prevention	2
92	RNA based vaccine	HDT-301: Self-replicating mRNA vaccine formulated as a lipid nanoparticle.	2	Day 0 + 28	IM	SENAI CIMATEC	1

IM: Intramuscular. IN: Intranasal. SC: Subcutaneous. ID: Intradermal.

Reference:

1. Organization, W.H., *Draft landscape and tracker of COVID-19 candidate vaccines*. 2021: who.int.
